# Apolipoproteins and the risk of giant cell arteritis—a nested case–control study

**DOI:** 10.1186/s13075-024-03273-1

**Published:** 2024-01-27

**Authors:** Karin Wadström, Lennart T. H. Jacobsson, Aladdin J. Mohammad, Kenneth J. Warrington, Eric L. Matteson, Carl Turesson

**Affiliations:** 1 Rheumatology, Department of Clinical Sciences, Malmö, Lund University, Malmö, 205 02 Sweden; 2 Center for Rheumatology, Academic Specialist Center, Stockholm, Region Stockholm Sweden; 3 Department of Rheumatology & Inflammation Research, Sahlgrenska Academy, University of Gothenburg, Gothenburg, Sweden; 4 Rheumatology, Department of Clinical Sciences, Lund, Lund University, Lund, Sweden; 5 Department of Rheumatology, Skåne University Hospital, Lund, Sweden; 6 Department of Medicine, University of Cambridge, Cambridge, UK; 7 Division of Rheumatology, Mayo Clinic College of Medicine and Science, Rochester, MN USA; 8 Department of Rheumatology, Skåne University Hospital, Malmö, Sweden

**Keywords:** Giant cell arteritis, Risk factors, Apolipoproteins, Lipids

## Abstract

**Background:**

The etiology of giant cell arteritis (GCA) and its predictors are incompletely understood. Previous studies have indicated reduced risk of future development of GCA in individuals with obesity and/or diabetes mellitus. There is limited information on blood lipids before the onset of GCA. The objective of the study was to investigate the relation between apolipoprotein levels and future diagnosis of GCA in a nested case–control analysis.

**Methods:**

Individuals who developed GCA after inclusion in a population-based health survey (the Malmö Diet Cancer Study; *N* = 30,447) were identified by linking the health survey database to the local patient administrative register and the national patient register. A structured review of medical records was performed. Four controls for every validated case, matched for sex, year of birth, and year of screening, were selected from the database. Anthropometric measures, self-reported physical activity, based on a comprehensive, validated questionnaire, and non-fasting blood samples had been obtained at health survey screening. Concentrations of apolipoprotein A-I (ApoA-I) and apolipoprotein B (ApoB) in stored serum were measured using an immunonephelometric assay. Potential predictors of GCA were examined in conditional logistic regression models.

**Results:**

There were 100 cases with a confirmed clinical diagnosis of GCA (81% female; mean age at diagnosis 73.6 years). The median time from screening to diagnosis was 12 years (range 0.3–19.1). The cases had significantly higher ApoA-I at baseline screening compared to controls (mean 168.7 vs 160.9 mg/dL, odds ratio [OR] 1.57 per standard deviation (SD); 95% confidence interval [CI] 1.18–2.10) (SD 25.5 mg/dL). ApoB levels were similar between cases and controls (mean 109.3 vs 110.4 mg/dL, OR 0.99 per SD; 95% CI 0.74–1.32) (SD 27.1 mg/dL). The ApoB/ApoA1 ratio tended to be lower in cases than in controls, but the difference did not reach significance. The association between ApoA-I and GCA development remained significant in analysis adjusted for body mass index and physical activity (OR 1.48 per SD; 95% CI 1.09–1.99).

**Conclusion:**

Subsequent development of GCA was associated with significantly higher levels of ApoA-I. These findings suggest that a metabolic profile associated with lower risk of cardiovascular disease may predispose to GCA.

**Supplementary Information:**

The online version contains supplementary material available at 10.1186/s13075-024-03273-1.

## Background

The etiology of giant cell arteritis (GCA) is not fully understood. Studies of predictors are therefore of major interest. A meta-analysis of observational studies demonstrated that patients with GCA have a significantly reduced prevalence of diabetes mellitus (DM) at the time of diagnosis [1], and a recent retrospective study from the Mayo Clinic reported a lower prevalence of DM at GCA diagnosis as well as 5 years prior to this date, compared to matched controls from the same catchment area [2]. A previous study by our group showed that cases who subsequently developed GCA had significantly lower fasting blood glucose, total cholesterol, and triglyceride levels compared to age- and sex-matched controls that did not develop GCA at a median of 20 years prior to diagnosis [3]. This study was based on the Malmö Preventive Medicine Project (MPMP), a population-based survey conducted between 1974 and 1992 in Malmö, Sweden, and identification of incident validated cases of GCA among participants in the MPMP. The observed negative association between statin treatment and incident biopsy-proven GCA in a regional study from southern Sweden [4] may reflect lower proportions with hyperlipidemia among people who develop GCA. Furthermore, several studies have indicated a negative association between body mass index (BMI) and development of GCA [2, 3, 5–8], in particular in women [6, 8]. Taken together, these observations implicate metabolic factors in the etiology of GCA.

There is limited information on blood lipids before the onset of GCA. In the population-based Reykjavik Study, total cholesterol levels did not differ between those who were subsequently diagnosed with GCA and those who were not [8]. In the study from the Mayo Clinic by Elfishawi et al., total cholesterol was higher compared to controls 5 years before GCA diagnosis, but not at 10 years prior to diagnosis [2]. This was largely driven by higher levels of high-density lipoprotein (HDL) cholesterol, whereas there was no significant difference in low density lipoprotein (LDL) cholesterol [2]. Triglycerides tended to be lower in patients with GCA, both before and at diagnosis, but the difference did not reach statistical significance [2]. At diagnosis, prior to treatment initiation, several studies have demonstrated reduced total cholesterol, LDL, and HDL in GCA [9, 10], possibly partly related to effects on lipid profiles by current inflammation.

Further knowledge on the dynamics of blood lipids in the pre-clinical phase of GCA may be gained from investigation of apolipoproteins. Whereas apolipoprotein B (ApoB) is a major protein component of LDL [11], apolipoprotein A-I (ApoA-I) has the corresponding role in HDL [12]. High levels of ApoB and low levels of ApoA-I have been shown to be better predictors of premature cardiovascular disease than LDL and HDL [13].

The objective of this study was to investigate the relation between plasma levels of cholesterol markers ApoA-I and ApoB and the risk of developing GCA. For this purpose, we performed a nested case–control analysis based on the population-based Malmö Diet Cancer Study (MDCS), using prospectively collected blood samples and data on other metabolic features.

## Methods

### Source population

The MDCS included residents of the city of Malmö, all men born 1923–1945, and all women born 1923–1950 (source population: *N* = 74,138). A total of 30,447 subjects (18,326 women and 12,121 men, average age 58 years) were screened between 1991 and 1996, corresponding to a 41% participation rate [14]. The only exclusion criteria were mental incapacity and insufficient Swedish language skills.

### Exposure information

Information on lifestyle factors, current and previous health status, and medications was collected by use of a self-administered questionnaire from all participants. Current occupation, as a measure of socioeconomic status, was classified as blue- vs white-collar work, based on self-reported job titles in the Swedish national census and standardized classification, as previously described [15]. Data on physical activity were based on a modified questionnaire adapted from the Minnesota Leisure Time Physical Activity Questionnaire [16], as previously described [17]. Participants were asked to estimate the number of minutes per week, for each of the four seasons, they spent performing 17 different physical activities. A physical activity score (PAS) was obtained by multiplying the answer with an intensity factor depending on the activity. This method has been validated against accelerometer-monitoring, in a subset of the present health survey population [18].

Anthropometric measures were obtained with the patient standing up. Height and weight were measured in light indoor clothing. Weight was recorded at intervals of 0.1 kg, and height was measured to the nearest centimeter. BMI was calculated as the ratio of weight (in kg) to height (in m)^2^. Waist was measured as the circumference (cm) between the lowest rib and the iliac crest. Hip circumference (cm) was measured as the largest circumference between waist and thighs. Waist/hip ratio was defined as the waist circumference divided by the hip circumference.

Blood pressure (BP) was measured once, after 10 min of rest in the supine position, by specially trained nurses, as previously described [19]. Hypertension was defined as being on antihypertensive treatment or having systolic BP/diastolic BP equal or greater than 140/90 mm Hg [19, 20].

Non-fasting blood samples were obtained at the time of inclusion in the health survey in a standardized manner and stored at – 80 °C. Serum concentrations of ApoA-I and ApoB were measured by Quest Diagnostics (San Juan Capistrano, CA), blinded to case–control status, using an immunonephelometric assay run on the Siemens BNII (Siemens, Newark, DE). The inter-assay variability was < 4.0% for both ApoA-I and ApoB [21].

### Cases and controls

Incident cases of GCA among participants in the MDCS were identified based on a registered diagnosis code of GCA in the National Patient Register for specialized inpatient and outpatient care or the local outpatient clinic administrative register for Malmö University Hospital after inclusion in the MDCS and through December 31, 2011. The medical records of the selected subjects were then reviewed in a structured process to ascertain the clinical diagnosis of GCA, with classification according to the 1990 American College of Rheumatology (ACR) criteria for GCA [22]. Some cases with typical clinical features were included, despite not fulfilling the classification criteria, based on expert opinion. In addition, initial dose of glucocorticosteroids, visual manifestations, large vessel involvement, and other disease characteristics were recorded.

Four controls for each validated case, matched for year of screening, year of birth, and sex, were randomly selected from the MDCS cohort. The controls were alive and living in Sweden and had not been diagnosed with GCA when the index subject received this diagnosis.

### Statistical analysis

Conditional logistic regression was used to examine potential predictors of GCA. For apolipoprotein levels, odds ratios (ORs) were calculated per standard deviation (SD) to enable comparison of effect sizes. To evaluate collinearity, correlations between apolipoprotein levels, BMI, and PAS were tested by Spearman’s rank test and Pearson’s test, as appropriate.

A sensitivity analysis was performed using only cases that fulfilled the 1990 ACR criteria for GCA and their matched controls.

## Results

There were 100 cases with a confirmed clinical diagnosis of GCA (Table 1). The mean age at diagnosis was 73.6 years, 81% were women, and the median time from screening to diagnosis was 12 years (interquartile range 8.5–15.5). Sixty-three had a positive temporal artery biopsy (TAB) and 92% fulfilled the ACR 1990 criteria. Patients that did not fulfill the classification criteria had missing data for some criteria items but a presentation and disease course compatible with GCA. Two patients had inconclusive TABs that were not repeated, two patients had no available ESR at diagnosis (but elevated CRP, 105 mg/l and 175 mg/l), one had acute ischemic optic neuropathy with positive TAB but no other signs of GCA, one had aortic involvement by imaging, but no TAB was performed, and one had missing information on the initial clinical examination.

**Table 1:** Characteristics of patients with giant cell arteritis at diagnosis

Cases, *n*	100
Female sex, *n* (%)	81 (81)
Age at GCA diagnosis, mean, years	73.6 (SD 6.0) (range 56.9–85.8)
Time from screening to GCA diagnosis, median, years	12.0 (IQR 8.5–15.5) (range 0.3–19.1)
Positive biopsy, *n*^a^	63 (63%)
Fulfilled ACR criteria, *n*	92 (92%)
Visual impairment at diagnosis, *n* (%)	41 (41%)
Large vessel involvement, *n* (%)	16 (16%)
ESR at time of diagnosis, mean^a^	80.7 (SD 31.5)
CRP at time of diagnosis, median^a^	89.0 (IQR 50.0–141.8)
Initial oral glucocorticosteroid dose, median, mg^b^	40 (IQR 20–60)

Data available for: ESR 91 cases, CRP 72 cases, initial dose of glucocorticosteroids 96 cases

*GCA* giant cell arteritis, *ACR* American College of Rheumatology, *ESR* erythrocyte sedimentation rate, *CRP* C-reactive protein, *SD* standard deviation, *IQR* interquartile rate

^a﻿^93 cases underwent temporal artery biopsy, 83 had a representative biopsy according to the pathology report

^b^﻿Prednisolone in all cases. Nine patients received intravenous glucocorticosteroids prior to oral prednisolone

The cases had higher ApoA-I at baseline screening compared to controls, whereas ApoB levels were similar between cases and controls (Table 2). The ApoB/ApoA-I ratio tended to be lower in cases than in controls, but the difference did not reach significance (Table 3). There was a significant association between ApoA1 and the risk of GCA in women, but not in men (Table 3). Cases had significantly lower BMI, only in women (Tables 2 and 3). There was a negative association for waist circumference with GCA in women and similar trend for the hip, resulting in a non-significant trend for the waist/hip ratio (Table 3). Waist or hip circumference had no significant impact on the risk of GCA in men (Table 3). There was a trend towards an association between higher PAS and GCA development in women, but not in men (Table 3). Smoking or hypertension did not have any major impact on the risk of GCA (Tables 2 and 3). A limited number of participants were on lipid lowering drug treatment at baseline, with numerically lower proportions among the cases (Table 1).

**Table 2:** Baseline characteristics of cases with later GCA diagnosis and controls, overall and stratified by sex

	**All**		**Women**		**Men**	
	Cases	Controls	Cases	Controls	Cases	Controls
***N***	100	400	81	324	19	76
**Age; mean, years (SD)**	62.3 (6.6)	61.9 (6.6)	62.3 (6.7)	61.9 (6.7)	62.2 (6.1)	61.7 (6.1)
**BMI; mean, kg/m**^**2**^** (SD)**	25.0 (4.1)	25.8 (3.9)	24.4 (3.3)	25.6 (4.0)	27.3 (6.2)	26.6 (3.5)
**Current smoking; *n*/*N* (%)**	16/92 (17)	93/397 (23)	14/73 (19)	79/321 (25)	2/19 (11)	14/76 (18)
**Hypertension *n*/*N* (%)**	63/100 (63)	274/398 (69)	55/81 (68)	220/323 (68)	8/19 (42)	54/75 (72)
**Lipid lowering drugs; *n* (%)**	2 (2)	17 (4)	2 (2)	12 (4)	0	5 (7)
**ApoA1; mean, mg/dL (SD)**	168.7 (29.1)	160.9 (24.4)	175.3 (27.0)	164.5 (23.6)	142.4 (21.6)	145.0 (21.1)
**ApoB; mean, mg/dL (SD)**	109.3 (24.3)	110.4 (27.8)	111.4 (23.9)	110.2 (29.0)	100.9 (24.5)	111.0 (21.9)
**ApoB/ApoA1; median (IQR)**	0.65 (0.53–0.78)	0.67 (0.56–0.82)	0.65 (0.53–0.75)	0.66 (0.55–0.79)	0.68 (0.55–0.93)	0.76 (0.62–0.89)
**Waist; median, cm (IQR)**	76.0 (71.0–85.8)	79.5 (72.0–88.8)	75.0 (69.5–79.0)	77.0 (70.0–85.0)	93.0 (83.0–107.0)	93.0 (86.0–101.0)
**Hip; median, cm (IQR)**	98.0 (91.0–101.0)	98.0 (93.0–104.0)	97.0 (91.0–100.0)	97.0 (92.0–104.0)	100.0 (97.0–105.0)	99.0 (95.0–105.0)
**Waist/hip; median (IQR)**	0.79 (0.76–0.84)	0.81 (0.77–0.87)	0.78 (0.75–0.81)	0.79 (0.75–0.83)	0.94 (0.88–0.98)	0.94 (0.91–0.97)
**Physical activity score median (IQR)**	6690 (4325–11,855)	5850 (4238–10,865)	6992 (4260–12,272)	6720 (4190 –10,530)	6180 4840–11,022	7862 (4666–12,877)

Information missing: ApoA, *n* = 8 (4 cases, 4 controls), ApoB, *n* = 8 (4 cases, 4 controls), physical activity, *n* = 11 (3 cases, 8 controls)

*ESR* erythrocyte sedimentation rate, *IQR* interquartile range, *SD* standard deviation

**Table 3:** Potential predictors of GCA in bivariate analyses using conditional logistic regression models, stratified by sex

	**All**		**Women**		**Men**	
	OR	95% CI	OR	95% CI	OR	95% CI
**BMI, per kg/m**^**2**^	0.93	0.87–1.00	0.89	0.82–0.97	1.05	0.92–1.20
**ApoA1**	1.57	1.18–2.10	1.80	1.30–2.48	0.84	0.42–1.66
**ApoB**	0.99	0.74–1.32	1.12	0.82–1.53	0.49	0.22–1.09
**ApoB/ApoA1**	0.79	0.59–1.07	0.81	0.58–1.13	0.75	0.40–1.38
**Waist**	0.76	0.55–1.04	0.64	0.44–0.93	1.20	0.66–2.18
**Hip**	0.81	0.62–1.05	0.74	0.55–1.00	1.16	0.65–2.09
**Waist/hip**	0.78	0.54–1.14	0.69	0.44–1.08	1.08	0.54–2.15
**Current smoking**						
No	1.00 (ref)		1.00 (ref)		1.00 (ref)	
Yes	0.66	0.34–1.28	0.70	0.34–1.46	0.89	0.04–20.0
**Physical activity score**	1.08	0.84–1.40	1.29	0.95–1.75	0.63	0.33–1.20

All odds ratios are per standard deviation unless otherwise indicated

Standard deviation values: ApoA1 25.5 mg/dL, ApoB 27.1 mg/dL, ApoB/ApoA1 0.22, waist 12.1 cm, hip 8.8 cm, waist/hip 0.08, physical activity score 5834

*BMI* body mass index, *OR* odds ratio, *CI* confidence interval, *SD* standard deviation

Correlations for apolipoproteins with BMI and physical activity score (Pearson’s test) were moderate (*r* ≤ 0.3). In analyses adjusted for BMI, and physical activity score, there was still an association between ApoA-I and GCA development (Table 4). In the fully adjusted model, including both BMI and physical activity score, the positive association between baseline ApoA-I levels and GCA remained significant (OR per SD 1.40; 95% CI 1.09–1.99). There was a consistent association between ApoA-I and GCA in women but a trend to the contrary in men (Table 4). The adjusted and unadjusted results for ApoB and ApoB/ApoA-I were similar (Tables 3 and 4).

**Table 4:** Potential predictors of giant cell arteritis in multivariate analyses, adjusted analyses

	**Adjusted for BMI**		**Adjusted for physical activity**		**Adjusted for BMI and physical activity**	
	OR	95% CI	OR	95% CI	OR	95% CI
**All**						
**ApoA1**	1.50	1.12–2.02	1.57	1.17–2.10	1.48	1.09–1.99
**ApoB**	1.01	0.76–1.35	1.04	0.78–1.40	1.08	0.81–1.46
**ApoB/ApoA1**	0.84	0.62–1.13	0.83	0.62–1.12	0.90	0.66–1.22
**Women**						
**ApoA1**	1.66	1.19–2.31	1.81	1.31–2.51	1.67	1.20–2.34
**ApoB**	1.18	0.86–1.62	1.15	0.83–1.58	1.22	0.88–1.69
**ApoB/ApoA1**	0.90	0.63–1.27	0.92	0.58–1.15	0.91	0.64–1.30
**Men**						
**ApoA1**	0.90	0.44–1.84	0.71	0.21–1.59	0.70	0.31–1.61
**ApoB**	0.47	0.21–1.07	0.61	0.28–1.33	0.60	0.27–1.33
**ApoB/ApoA1**	0.70	0.37–1.32	0.87	0.47–1.63	0.86	0.46–1.64

All odds rations are per standard deviation: ApoA1 25.5 mg/dL, ApoB 27.1 mg/dL, ApoB/ApoA1 0.22

*BMI* body mass index, *OR* odds ratio, *CI* confidence interval

In the sensitivity analysis including only cases that developed GCA that fulfilled the 1990 ACR criteria and their matched controls, descriptive data (Supplementary Table 1) and results of unadjusted conditional logistic regression (Supplementary Table 2) were similar to the main analysis, with the exception of the negative association between waist circumference and GCA, which reached significance in this analysis.

## Discussion

In this study of individuals who subsequently developed GCA and controls who did not, we observed a significant association between serum ApoA1 levels and subsequent GCA. This association was restricted to women and not explained by differences in BMI or physical activity. By contrast, ApoB levels or the ApoB/ApoA1 ratio were not predictive of GCA.

To our knowledge, this is the first study to examine apolipoproteins prior to GCA onset. The results are compatible with previous findings of an increased risk of GCA in women with a healthy metabolic profile. Specifically, they are in agreement with the retrospective study by Elfishawi et al., which reported higher HDL levels compared to controls five years before diagnosis, with a similar trend 10 years prior to diagnosis [2].

In general, ApoA-I is considered to have mainly anti-inflammatory properties [12]. In this context, it may be relevant as a marker of a metabolic profile that predisposes to GCA, rather than being directly involved in the early pathogenesis of vasculitis.

It has been suggested that increased availability of glucose metabolites, as in DM or pre-DM, may contribute to mechanisms that are protective from GCA by limiting T cell dysregulation. The immune checkpoint that involves the programmed death receptor-1 (PD-1) and its interaction with its ligand PD-L1, which is dysfunctional in GCA [23], can be affected by glucose metabolites. Increased availability of mitochondrial pyruvate has been shown to lead to upregulation of PD-L1 on macrophages [24].

The concept of early metabolic regulation of mechanisms leading to GCA is of particular interest, as investigation of plasma proteins related to inflammation in the same patient sample revealed higher circulating levels of IFN-γ and other T cell-related markers years before the clinical onset of GCA, supporting an extended pre-clinical phase in GCA [25]. Interestingly, IL-6 was not elevated prior to GCA, suggesting that effects of IL-6 on the production of apolipoproteins in the liver should not affect the analyses performed in the present study.

The observed patterns differ from that previously reported by our group on rheumatoid arthritis (RA), where there was a significant association between higher cholesterol levels and subsequent development of RA in women [26]. This suggests fundamentally different relations between lipid metabolism and pathophysiology for RA and GCA, two of the more common inflammatory diseases in the field of rheumatology.

Limitations of this study include the fact that apolipoproteins were only measured at one time point in the MDCS. Therefore, we were not able to examine the relation between longitudinal changes in ApoB and ApoA-I and development of GCA. The low number of individuals on lipid lowering drugs at baseline indicate that such treatment did not have a major impact on the reported apolipoprotein levels. However, we cannot exclude that subsequent statin treatment may have affected the risk of GCA. Furthermore, the number of male subjects in this study was limited, and the results from this subset should be interpreted with caution.

The use of samples that have been stored for an extended time could potentially be an issue. Apolipoprotein levels have been shown to be very stable over time in frozen samples stored for 1 year [27], but there is limited data on the impact of storage for longer time. As cases and controls were matched for year of inclusion in the MDCS, and hence for storage time, this should not lead to a biased comparison.

The present sample of individuals who subsequently developed GCA partly overlaps with that included in our previous study of total cholesterol levels prior to GCA diagnosis [3], as 38 of the pre-GCA cases included in the present study had previously been included in the MPMP. However, inclusion in the MPMP and the MDCS were separate and in most cases several years apart. Finally, as studies of metabolic features as potential risk factors of GCA have mainly been performed in populations from Scandinavia [3, 5, 6, 8] or in Minnesota, USA [2], which has a population with predominance of white Caucasians with Northern European/Scandinavian ancestry, we cannot exclude that associations would differ in other ethnic groups.

Strengths of the present study include the population-based design, with prospectively collected information from a representative sample of residents in the area, and the independent identification of individuals with a subsequent development of GCA. Furthermore, other important measures such as BMI and physical activity were available, and the significant association with ApoA-I in adjusted analyses suggests that specific metabolic pathways may explain the present results.

## Conclusion

Subsequent development of GCA was associated with significantly higher levels of Apo-AI, and there was a trend towards lower ApoB/ApoA1 ratio and GCA risk. Taken together with previous observations on lower BMI and lower fasting glucose levels in individuals who subsequently develop GCA, these findings support the concept of an association between a healthier metabolic profile that does not predispose to cardiovascular disease and increased risk of GCA.

### Supplementary Information

**Additional file 1: Supplementary Table 1.** Baseline characteristics of cases with later GCA diagnosis who fulfilled the 1990 American College of Rheumatology classification criteria and matched controls

**Additional file 2: Supplementary Table 2.** Potential predictors of giant cell arteritis in bivariate analyses using conditional logistic regression models. Sensitivity analysis, restricted to cases who fulfilled the 1990 American College of Rheumatology classification criteria, and matched controls.

## Data Availability

A limited and fully anonymized dataset containing the data that support the main analyses is available from the corresponding author on reasonable request.

## Abbreviations

ApoA-I: Apolipoprotein A-I
ApoB: Apolipoprotein B
BMI: Body mass index
BP: Blood pressure
DM: Diabetes mellitus
GCA: Giant cell arteritis
HDL: High density lipoproteins
LDL: Low density lipoproteins
MDCS: Malmö Diet and Cancer Study
MPMP: Malmö Preventive Medicine Project
OR: Odds ratio
PAS: Physical activity score
PD-1: Programmed death receptor 1
PD-L1: Programmed death ligand 1
RA: Rheumatoid arthritis
SD: Standard deviation
