# Supplementary material for: Apolipoproteins and the risk of giant cell arteritis—a nested case–control study
Source: Arthritis Res Ther. 2024 Jan 27;26:37. doi: 10.1186/s13075-024-03273-1 (PMC10821258; doi:10.1186/s13075-024-03273-1)
Supplement: Supplementary file 1 — Additional file 1: Supplementary Table 1. Baseline characteristics of cases with later GCA diagnosis who fulfilled the 1990 American College of Rheumatology classification criteria and matched controls [file 13075_2024_3273_MOESM1_ESM.docx]

**Supplementary Table 1.** Baseline characteristics of cases with later GCA diagnosis who fulfilled the 1990 American College of Rheumatology classification criteria and matched controls

|  | **Cases** | **Controls** |
| --- | --- | --- |
| **N** | 92 | 368 |
| **Age; mean, years (SD)** | 62.6 (6.3) | 62.1 (6.3) |
| **BMI; mean, kg/m^2^ (SD)** | 24.9 (4.0) | 25.8 (3.9) |
| **Current smoking; n/N (%)** | 14/88 (16) | 73/366 (20) |
| **Hypertension n/N (%)** | 62/92 (67) | 256/366 (70) |
| **Lipid lowering drugs; n (%)** | 2 (2) | 14 (4) |
| **ApoA1; mean, mg/dL (SD)** | 169.0 (28.7) | 161.0 (24.6) |
| **ApoB; mean, mg/dL (SD)** | 108.8 (24.1) | 110.6 (27.8) |
| **ApoB/ApoA1; median (IQR)** | 0.65 (0.52 - 0.78) | 0.68 (0.56 - 0.82) |
| **Waist; median, cm (IQR)** | 76 (71 - 86) | 80 (72 - 90) |
| **Hip; median, cm (IQR)** | 98 (91 - 101) | 98 (93 - 104) |
| **Waist/hip; median (IQR)** | 0.79 (0.76 - 0.85) | 0.81 (0.76 - 0.87) |
| **Physical activity score median (IQR)** | 6 585 (4 320 – 10 693) | 7 094 (4 320 - 10 830) |

*ESR* erythrocyte sedimentation rate, *IQR* interquartile range, *SD* standard deviation

Information missing: ApoA1: n=8 (cases 4, controls 4), ApoB: n=8 (cases 4, controls 4), Physical activity n=8 (3 cases, 5 controls).
