# Supplementary material for: Apolipoproteins and the risk of giant cell arteritis—a nested case–control study
Source: Arthritis Res Ther. 2024 Jan 27;26:37. doi: 10.1186/s13075-024-03273-1 (PMC10821258; doi:10.1186/s13075-024-03273-1)
Supplement: Supplementary file 2 — Additional file 2: Supplementary Table 2. Potential predictors of giant cell arteritis in bivariate analyses using conditional logistic regression models. Sensitivity analysis, restricted to cases who fulfilled the 1990 American College of Rheumatology classification criteria, and matched controls. [file 13075_2024_3273_MOESM2_ESM.docx]

**Supplementary Table 2.** Potential predictors of giant cell arteritis in bivariate analyses using conditional logistic regression models. Sensitivity analysis, restricted to cases who fulfilled the 1990 American College of Rheumatology classification criteria, and matched controls.

|  | | **OR** | **95 % CI** |
| --- | --- | --- | --- |
| **BMI, per kg/m^2^** | | 0.91 | 0.84 - 0.98 |
| **ApoA1** | | 1.58 | 1.18 - 2.09 |
| **ApoB** | | 0.96 | 0.72 - 1.28 |
| **ApoB/ApoA1** | | 0.76 | 0.56 - 1.03 |
| **Waist** | | 0.65 | 0.46 - 0.93 |
| **Hip** | | 0.75 | 0.56 - 1.01 |
| **Waist/hip** | | 0.66 | 0.44 - 1.00 |
| **Current smoking** | no | 1.00 (ref) |  |
|  | yes | 0.75 | 0.37 - 1.53 |
| **Physical activity score** | | 0.96 | 0.73 - 1.26 |

*BMI* body mass index, *OR* odds ratio, *CI* confidence interval, *SD* standard deviation

All odds ratios are per standard deviation unless otherwise indicated

Standard deviation values: ApoA1 25.6 mg/dL, ApoB 27.2 mg/dL, ApoB/ApoA1 0.21, waist 12.0 cm, Hip 8.6 cm, waist/hip 0.08, physical activity score 5868
